# Supplementary material for: The Manufacturing Process of Lotus (Nelumbo Nucifera) Leaf Black Tea and Its Microbial Diversity Analysis
Source: Foods. 2025 Feb 6;14(3):519. doi: 10.3390/foods14030519 (PMC11817234; doi:10.3390/foods14030519)
Supplement: Supplementary file 1 [file foods-14-00519-s001.zip › foods-3397845-supplementary.pdf]

Supplemental Figure Caption

Figure S1. Fungal taxonomic compositions showing the microbial successions at species level. The results showed the top10 species and the other species were classified into "Others". WRL, withering and rolled leaves; FL3, leaves fermented for 3 h; FL6, leaves fermented for 6 h; FL12, leaves fermented for 12 h; FL24, leaves fermented for 24 h.

Figure S2. Interaction networks in the microbial communities at the generic level during the fermentation process of lotus leaves. bacterial (A), Fungal (B).

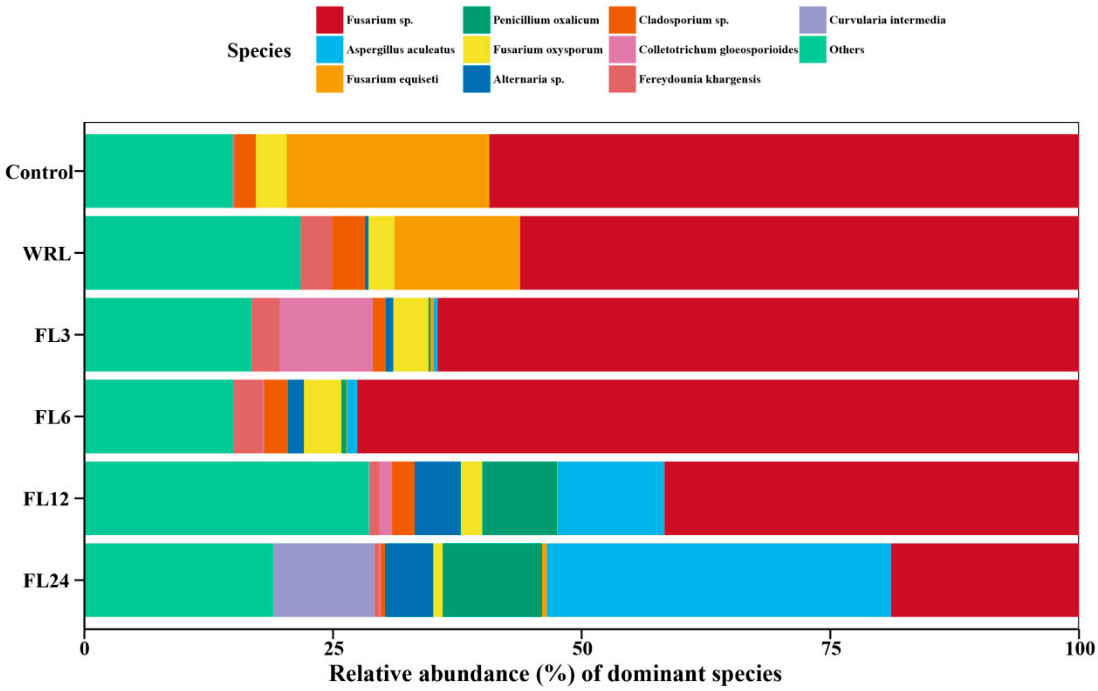
Species
Fusarium sp.
Aspergillus aculeatus
Fusarium equiseti
Penicillium oxalicum
Fusarium oxysporum
Alternaria sp.
Cladosporium sp.
Colletotrichum gloeosporioides
Fereydounia khargensis
Curvularia intermedia
Others
Control
WRL
FL3
FL6
FL12
FL24
0
25
50
75
100
Relative abundance (%) of dominant species

Figure S1

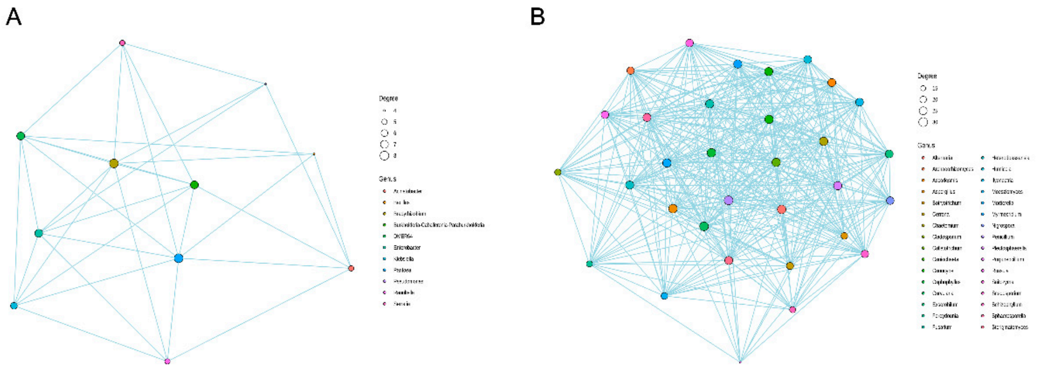
A
B
Degree
4
5
6
7
8
Genus
Enterobacter
Pantoea
Degree
Genus
Chaetomium
Cladosporium
Fusarium

Figure S2

Table S1. Polyphenol oxidase and antioxidant activity of lotus green tea.

	Polyphenol oxidase activity (U)	Antioxidant activity		
		ABTS (mg Vc/g)	DPPH (mg Vc/g)	Hydroxyl radical scavenging activity (mg Vc/g)
Lotus green tea	0.12 ± 0.01	8.07 ± 1.30	45.01 ± 0.39	10.58 ± 3.31

Values were expressed as the mean ± standard deviation (SD).

Vc, Vitamin C.
